# Effect of High-quality Nursing on Improvement of Anxiety and Depression of Patients with Acute Stroke in MRI Examination

**Published:** 2017-12

**Authors:** Lin WU, Li ZHANG

**Affiliations:** 1. Dept. of MRI, Liaocheng People’s Hospital, Liaocheng, Shandong, China; 2. Dept. of Cardiology, Liaocheng People’s Hospital, Liaocheng, Shandong, China

**Keywords:** High-quality nursing, Acute stroke, MRI examination, Anxiety, Depression

## Abstract

**Background:**

We aimed to evaluate the effect of high-quality nursing on improvement of anxiety and depression of patients with acute stroke in magnetic resonance imaging (MRI) examination.

**Methods:**

A total of 120 patients diagnosed as acute stroke for the first time were enrolled in Liaocheng People’s Hospital from 2016–2017 and randomly divided into control group (n=60) and observation group (n=60). All patients received cerebral MRI examination at 6h and 24h after admission and before discharge. The control group was treated with routine nursing, while the observation group was treated with high-quality nursing, and the specific nursing measures included the establishment of high-quality nursing group, full evaluation of the severity of disease, timely solving of difficulties in MRI examination, understanding of the patient’s anxiety and depression, establishment of personal information files before discharge, etc. The completion rate and average duration of examination, the improvement of anxiety and depression and the nursing satisfaction were compared between the two groups.

**Results:**

In observation group, the completion rate of MRI examination was significantly increased (*P*=0.035), the average duration was shortened (*P*=0.011), the anxiety and depression scores (self-rating anxiety scale (SAS) and self-rating depression scale (SDS)) were improved obviously (*P*=0.006 and 0.009), and the nursing satisfaction score and rate was increased (*P*=0.000 and 0.027); the differences were statistically significant (*P*<0.05).

**Conclusion:**

High-quality nursing can significantly improve the anxiety and depression of patients with acute stroke in MRI examination, which has a better application value in increasing the completion rate of examination, shortening the duration of examination and improving the nursing satisfaction.

## Introduction

Acute stroke is one of the important types of cerebrovascular diseases threatening the life and health of patients, including hemorrhagic stroke and ischemic stroke. Determining the target vessel, cerebral position and severity of surrounding important nerve and vascular injury as soon as possible is of great significance in evaluating the disease, developing the treatment strategies and predicting the clinical outcome (1).

Magnetic resonance imaging (MRI) is characterized by extremely high soft tissue resolution, spatial and temporal resolution, multiple sequences and post-processing techniques, non-invasion and non-radiation, etc., so it is often used as a preferred examination method in the diagnosis of stroke (2). However, the state of stroke changes quickly, and repeated examination is often needed to understand the progression of disease. Besides, the MRI examination takes a long time, and patients need to stay in the MRI machine in a relatively dark and closed environment for brain examination (3); at the same time, MRI produces huge noise, which is easy to increase the patient’s negative emotions, such as tension, anxiety and depression (4, 5).

Moreover, acute stroke also leads to low spirits, partial loss of self-care ability, cognitive dysfunction, and serious decline in the life quality of patients easily, and it can make patients produce the world-weary and pessimistic attitudes towards life (6, 7). Therefore, increasing the nursing quality of MRI examination has a very important practical significance in optimizing the examination efficiency and increasing the patient’s compliance.

This study mainly analyzed the effect of high-quality nursing on improvement of anxiety and depression of patients with acute stroke in MRI examination.

## Methods

### Object data

A total of 120 patients diagnosed as acute stroke for the first time in Liaocheng People’s Hospital from January 2016 to January 2017 were continuously selected. Signed written informed consents were obtained from all participants before the study. Inclusion criteria: 1) patients meeting the diagnostic criteria of acute stroke; 2) patients who completed the MRI examination at different time points according to the requirements of research; 3) patients with complete clinical data and who signed the informed consent. Exclusion criteria: 1) patients complicated with other diseases and receiving other examinations; 2) patients who were not allowed to complete the MRI examination due to severe disease; 3) patients with contraindications of MRI examination; 4) patients with a history of neuropsychiatric disease, severe anxiety or depression.

Informed consents were signed by the patients and/or guardians. The study was approved by the ethics committee of Liaocheng People’s Hospital. The patients were divided into control group (n=60) and observation group (n=60) using a random number table. In control group, there were 38 males and 22 females aged 45–78 years ((56.5±12.3) years old on average); the onset time of disease was 1–6h ((3.5±1.2)h on average); the National Institutes of Health stroke Scale (NIHSS) score was 15–32 points ((24.5±8.7) points on average); the duration of education was 10–18 years ((13.5±3.6) years on average); there were 20 cases of hypertension, 8 cases of diabetes mellitus and 6 cases of coronary heart disease. In observation group, there were 40 males and 20 females aged 44–79 years ((55.8±13.6) years old on average); the onset time of disease was 2–5.5h ((3.3±1.1) h on average); the NIHSS score was 13–30 points ((22.8±8.2) points on average); the duration of education was 8–17 years ((12.9±3.4) years on average); there were 16 cases of hypertension, 10 cases of diabetes mellitus and 7 cases of coronary heart disease. The baseline data of the two groups were comparable (*P*>0.05).

### Research methods

Patients in the two groups received cerebral MRI examination at 6h and 24h after admission and before discharge. PHILIPS 3.0T superconducting magnetic resonance scanner and supporting software were used for T1, T2 and DWI sequence scanning, followed by diagnosis in the postprocessing station. The sequence scanning parameters were the same in the two groups of patients, and the scanning was performed by the same group of experienced scanning and diagnosis physicians.

The control group was treated with routine nursing. According to the doctor’s advice, the examination time and order were determined, as well as communication with the patients and their families, and preparations of examination, such as the safe movement of treatment bed, placement of bedside monitor, emergency drug; 1–2 nurses were responsible for one patient. The observation group was treated with high-quality nursing, and the specific nursing measures included the establishment of high-quality nursing group, full evaluation of the severity of disease, timely solving of difficulties in MRI examination, understanding of the patient’s anxiety and depression, and establishment of personal information files before discharge, etc. The high-quality nursing group consisted of 1 head nurse, 2 senior nurses and 4–6 junior nurses. The head nurse was responsible for supervising the implementation of high-quality nursing and the deficiencies in practical work and proposing the improvement measures. The senior nurses were responsible for the training and assessment of junior nurses. The vital signs of patients were fully evaluated, the examination time and order was reasonably determined, and nursing staffs at different levels were assigned, combined with requirements of different patient. Risk stratification was performed for patients, and one supervisor nurse (working experience for at least 5 years and ability to deal with all emergency and severe diseases) and two higher nurses (working experience for at least 2 years and ability to skillfully perform invasive operation) were assigned for patient with extremely unstable conditions. The clinical nursing pathway was developed, and all kinds of nursing services were shown in the table, facilitating the nursing operation and quality improvement; the safety education was strengthened for patients and their families, and the changes in disease should be found in time; the service attitude was improved to understand patient’s psychological negative emotions, and help eliminate the anxiety or depression; finally, a warm, harmonious and clean environment in wards was created, and the different needs of patients were addressed.

### Observational indexes

The completion rate and average duration of examination, the improvement of anxiety and depression before discharge and the nursing satisfaction before discharge were compared between the two groups. The duration of examination was from the doctor’s advice to the end of safe examination, including the time from the doctor’s advice to the readiness of examination outside, the time from safely leaving the ward to entering the MRI room, and the time from safely being transferred onto the MRI examination bed, completion of examination to safely returning to the ward. The examination should be completed and the results could be used for analysis; all the complications should be eliminated in the process for the safety of examination outside. Self-rating anxiety scale (SAS) and self-rating depression scale (SDS) were used for the evaluation of anxiety and depression; the total score is 0–100 points, and the higher the score is, the severer the symptoms will be. The nursing satisfaction was evaluated using the self-made questionnaire of our hospital, including the nursing operation, nursing attitude, examination outside and problem solving; the total score is 0–100 points, the higher the score is, the better nursing satisfaction will be; 80–100 points: more satisfactory; 60–79 points: general; 0–59 points: unsatisfactory. The reliability and validity test proved that it had a better clinical application value.

### Statistical analysis

Statistical analysis was performed using Statistical Product and Service Solutions (SPSS) 20.0 software (SPSS Inc., Chicago, IL, USA). Measurement data were presented as mean ± standard deviation; independent-samples *t* test was used for the intergroup comparison, and paired *t* test was used for the intragroup comparison. Enumeration data were presented as case or percentage (%), and chi-square test was used for the intergroup comparison. *P*<0.05 suggested that the difference was statistically significant.

## Results

In observation group, the completion rate of examination was increased (*P*=0.035), and the average duration was shortened (*P*=0.011); the differences were statistically significant (Table 1).

**Table 1:** Comparisons of completion rate and average duration of examination between the two groups (n (%))

***Group***	***n***	***One-time examination***	***Two-time examination***	***Three-time examination***	***Duration of examination (h)***
Control group	60	6 (10.0)	14 (23.3)	40 (66.7)	2.9±0.8
Observation group	60	2 (3.3)	8 (13.3)	50 (83.3)	2.2±0.5
t/χ^2^				4.444	5.236
*P*				0.035	0.011

There were no significant differences in SAS and SDS scores between the two groups before examination (*P*= 0.628 and 0.724, respectively), and the scores in observation group before discharge had no differences compared with those before examination (*P*=0.876), but the scores in control group before discharge were significantly higher than those before examination (*P*=0.000); the SAS and SDS scores in observation group before discharge were obviously lower than those in control group, and the differences were statistically significant (*P*=0.006 and 0.009, respectively) (Table 2).

**Table 2:** Comparisons of anxiety and depression between the two groups

***Group***	***n***	***SAS***		***SDS***	
		Before examination	Before discharge	Before examination	Before discharge
Control group	60	25.6±4.8	30.8±5.2#	28.3±5.3	33.5±6.3#
Observation group	60	26.7±4.6	23.4±4.3	29.6±5.5	28.4±5.1
*t*		0.325	5.865	0.269	5.724
*P*		0.628	0.006	0.724	0.009

Note:

# , comparisons of SAS and SDS scores in control group before discharge with those before examination, *P*<0.05.

The nursing satisfaction score in observation group was increased, and the satisfaction rate was also increased; the differences were statistically significant (*P*=0.000 and 0.027, respectively) (Table 3).

**Table 3:** Comparison of nursing satisfaction between the two groups (n (%))

***Group***	***n***	***Nursing satisfaction score***	***More satisfactory***	***General***	***Unsatisfactory***
Control group	60	79.8±13.4	42 (70.0)	15 (25.0)	3 (5.0)
Observation group	60	86.9±12.3	52 (86.7)	7 (11.7)	1 (1.7)
*t*/χ^2^		6.532	4.910		
*P*		0.000	0.027		

## Discussion

At present, study on the nursing of patients with stroke in MRI examination, and how to improve the nursing quality has an important application value for the early correct diagnosis, evaluation and prognosis of stroke is rare. According to statistics (8), the incidence rate of early anxiety or depression after stroke is up to 50–80%, and it may be higher in rehabilitation period. Adverse psychological mood is both the outcome of stroke and an important adverse factor affecting the treatment and prognosis of stroke (9). The impact of MRI, as the major examination method of early stroke, on the negative emotions of patients cannot be ignored, especially anxiety or depression, while exerting its superiority (10). The incidence of anxiety or depression for normal people in one-time MRI examination is about 0.5–5.0%, and it can increase to 10–30% in the three-time MRI examination and above, regardless of the interval time (11). There are few objective data about the anxiety or depression of patients with stroke receiving multiple MRI examinations. Therefore, this study had some innovation.

The results of this study showed that in observation group, the completion rate of MRI examination was significantly increased, the average duration was shortened, the anxiety and depression scores were improved obviously, and the nursing satisfaction was increased; the differences were statistically significant (*P*<0.05), suggesting that high-quality nursing can significantly improve the anxiety and depression of patients with acute stroke in MRI examination, which has a better application value in increasing the completion rate of examination, shortening the duration of examination and improving the nursing satisfaction. High-quality nursing reflects the improvement of quality of traditional nursing, which can make the best use of limited nursing resources, fully mobilize the sense of responsibility and enthusiasm of nurses, strengthen the professional nursing skills and improve the nursing service level (12). Moreover, the focuses in nursing service include improving the skills of nursing operations, service attitude and problem-solving ability (13); getting familiar with the examination process and possible contingencies combined with the requirements of MRI examination (14); fully understanding the different psychological fluctuations of patients, safety management in examination outside, and performing a variety of examinations scientifically and effectively (15). At the same time, the health education for patients and their families should be strengthened to give full play to their active participation (16).

However, there are many interference factors and difficulties in the examination outside in the practical work. For example, patients and their families do not understand the examination and think that it is not necessary or is a threat to life, so doctors should explain the examination in patience and get the permission (17); in addition, there are many unsafe factors in the examination outside, such as many early complications, unstable vital signs, longer distance, fewer operating window, longer diagnosis waiting time and poor examination environment (18, 19). At this point, high-quality nursing is an important prerequisite for the smooth completion of examination (20). Moreover, there is no uniform provision on the best time of examination outside, or unified understanding of the importance of examination outside.

## Conclusion

High-quality nursing can significantly improve the anxiety and depression of patients with acute stroke in MRI examination, which has a better application value in increasing the completion rate of examination, shortening the duration of examination and improving the nursing satisfaction. The observational indexes in this study still lacked a uniform standard, and the sample size was small, so it still needs to be verified by further studies.

## Ethical considerations

Ethical issues (Including plagiarism, informed consent, misconduct, data fabrication and/or falsification, double publication and/or submission, redundancy, etc.) have been completely observed by the authors.

## References

[1] Zhang Y Li KS Ning YZ Fu CH Liu HW Han X Cui FY Ren Y Zou YH (2016). Altered structural and functional connectivity between the bilateral primary motor cortex in unilateral subcortical stroke: A multimodal magnetic resonance imaging study. Medicine (Baltimore), 95: e4534. 27495109 10.1097/MD.0000000000004534 PMC4979863

[2] Lazaridou A Astrakas L Mintzopoulos D Khanicheh A Singhal AB Moskowitz MA Rosen B Tzika AA (2013). Diffusion tensor and volumetric magnetic resonance imaging using an MR-compatible hand-induced robotic device suggests training-induced neuroplasticity in patients with chronic stroke. Int J Mol Med, 32: 995–1000. 23982596 10.3892/ijmm.2013.1476 PMC3820572

[3] Hutton J Walker LG Gilbert FJ Evans DG Eeles R Kwan-Lim GE Thompson D Pointon LJ Sharp DM Leach MO (2011). Psychological impact and acceptability of magnetic resonance imaging and X-ray mammography: The MARIBS Study. Br J Cancer, 104: 578–586. 21326245 10.1038/bjc.2011.1 PMC3049597

[4] Peng J Zhou J Wu X (2016). Dual-domain denoising in three dimensional magnetic resonance imaging. Exp Ther Med, 12: 653–660. 27446257 10.3892/etm.2016.3345 PMC4950751

[5] Bangard C Paszek J Berg F Eyl G Kessler J Lackner K Gossmann A (2007). MR imaging of claustrophobic patients in an open 1.0T scanner: Motion artifacts and patient acceptability compared with closed bore magnets. Eur J Radiol, 64: 152–157. 17374468 10.1016/j.ejrad.2007.02.012

[6] Gyagenda JO Ddumba E Odokonyero R Kaddumukasa M Sajatovic M Smyth K Katabira E (2015). Post-stroke depression among stroke survivors attending two hospitals in Kampala Uganda. Afr Health Sci, 15: 1220–1231. 26958024 10.4314/ahs.v15i4.22 PMC4765432

[7] Shi MC Chu FN Li C Wang SC (2017). Thrombolysis in acute stroke without angiographically documented occlusion. Eur Rev Med Pharmacol Sci, 21: 1509–1513. 28429356

[8] Kronenberg G Gertz K Heinz A Endres M (2014). Of mice and men: Modelling post-stroke depression experimentally. Br J Pharmacol, 171: 4673–4689. 24838087 10.1111/bph.12775 PMC4209937

[9] Thomas SA Coates E Das NR (2016). Behavioural Activation Therapy for Depression after Stroke (BEADS): A study protocol for a feasibility randomised controlled pilot trial of a psychological intervention for post-stroke depression. Pilot Feasibility Stud, 2: 45. 27965862 10.1186/s40814-016-0072-0 PMC5153669

[10] Dewey M Schink T Dewey CF (2007). Frequency of referral of patients with safety-related contraindications to magnetic resonance imaging. Eur J Radiol, 63: 124–127. 17383136 10.1016/j.ejrad.2007.01.025

[11] Dewey M Schink T Dewey CF (2007). Claustrophobia during magnetic resonance imaging: Cohort study in over 55,000 patients. J Magn Reson Imaging, 26: 1322–1327. 17969166 10.1002/jmri.21147

[12] Park SC Lee HY Lee DW (2015). Knowledge and Attitude of 851 Nursing Personnel toward Depression in General Hospitals of Korea. J Korean Med Sci, 30: 953–959. 26130960 10.3346/jkms.2015.30.7.953 PMC4479951

[13] Enders J Zimmermann E Rief M (2011). Reduction of claustrophobia with short-bore versus open magnetic resonance imaging: A randomized controlled trial. Plos One, 6: e23494. 21887259 10.1371/journal.pone.0023494 PMC3161742

[14] Katznelson R Djaiani GN Minkovich L Fedorko L Carroll J Borger MA Cusimano RJ Karski J (2008). Prevalence of claustrophobia and magnetic resonance imaging after coronary artery bypass graft surgery. Neuropsychiatr Dis Treat, 4: 487–493. 18728736 10.2147/ndt.s2699 PMC2518378

[15] Okorie CK Ogbole GI Owolabi MO Ogun O Adeyinka A Ogunniyi A (2015). Role of diffusion-weighted imaging in acute stroke management using low-field magnetic resonance imaging in resource-limited settings. West Afr J Radiol, 22: 61–66. 26709342 10.4103/1115-3474.162168 PMC4689208

[16] Moustafa RR Baron JC (2007). Clinical review: Imaging in ischaemic stroke--implications for acute management. Crit Care, 11: 227. 17875224 10.1186/cc5973 PMC2556770

[17] Liebeskind DS Alexandrov AV (2012). Advanced multimodal CT/MRI approaches to hyperacute stroke diagnosis, treatment, and monitoring. Ann N Y Acad Sci, 1268: 1–7. 22994214 10.1111/j.1749-6632.2012.06719.x PMC4144352

[18] Farr TD Wegener S (2010). Use of magnetic resonance imaging to predict outcome after stroke: A review of experimental and clinical evidence. J Cereb Blood Flow Metab, 30: 703–717. 20087362 10.1038/jcbfm.2010.5 PMC2949172

[19] Walter S Kostpopoulos P Haass A (2010). Bringing the hospital to the patient: First treatment of stroke patients at the emergency site. Plos One, 5(10): e13758. 21060800 10.1371/journal.pone.0013758 PMC2966432

[20] Parody E Pedraza S Garcia-Gil MM Crespo C Serena J Davalos A (2015). Cost-Utility analysis of magnetic resonance imaging management of patients with acute ischemic stroke in a Spanish hospital. Neurol Ther, 4: 25–37. 26847673 10.1007/s40120-015-0029-x PMC4470974

